# Study on the Effect of Zinc on the Rheological, Mechanical and Thermal Properties and Fire Hazard of Unfilled and Filled CR/BR Vulcanizates

**DOI:** 10.3390/polym12122904

**Published:** 2020-12-03

**Authors:** Aleksandra Smejda-Krzewicka, Anna Słubik, Krzysztof Strzelec, Przemysław Rybiński

**Affiliations:** 1 Institute of Polymer and Dye Technology, Lodz University of Technology, Stefanowskiego 12/16, 90-924 Lodz, Poland; anna.slubik@dokt.p.lodz.pl (A.S.); krzysztof.strzelec@p.lodz.pl (K.S.); 2 Institute of Chemistry, Jan Kochanowski University, Zeromskiego 5, 25-369 Kielce, Poland; przemyslaw.rybinski@ujk.edu.pl

**Keywords:** chloroprene rubber, butadiene rubber, zinc, flammability, cone calorimetry

## Abstract

This paper discusses the cross-linking and functional properties of elastomer composites containing chloroprene rubber (CR) and butadiene rubber (BR) cured in the presence of zinc (Zn) and reinforced with mineral fillers. The research aimed to evaluate the effectiveness of zinc as a new cross-linking substance with the simultaneous production of elastomer materials with good mechanical properties and a reduced fire hazard. The article concerns the study and explanation of the dependencies influencing the processing and functional properties of unfilled or filled elastomer blends containing different elastomers ratio or different zinc’s amount. The following fillers were used: silica, kaolin, chalk and montmorillonite. The results revealed that the cross-linking degree of CR/BR blends decreased with the increasing amount of butadiene rubber in the blends. The mechanical properties of the cured blends depended on the proportion of elastomers in the composites, the zinc amount, and the presence and type of filler. The flammability of CR/BR/Zn vulcanizates has been investigated before and after the filling. The parameters assessed by the oxygen index method and cone calorimetry, characterizing the behavior of the tested CR/BR/Zn vulcanizates under fire conditions, have shown that they constitute a low fire hazard and can be considered as non-flammable materials.

## 1. Introduction

Butadiene rubber (BR) is one of the most important synthetic elastomers, obtained by the polymerization of 1,3-butadiene [1,2,3]. Depending on the polymerization method and the catalysts used (Ziegler–Natta catalysts), polymers with a predominance of *cis*-1.4, *trans*-1.4 or *vinyl*-1.2 structures can be obtained in this process. The participation of individual units is important for the chemical structure of BR, and thus for its functional properties [4,5]. Vulcanizates made of stereospecific BR (showing a high regularity of structure) have a low hysteresis, and thus a low coefficient of friction, high abrasion resistance and good dynamic properties [6,7,8]. Additionally, due to its low glass transition temperature (*T*_g_ ~105 °C), compared to the styrene-butadiene rubber (SBR) or natural rubber (NR), this rubber is preferred, in particular, for the production of tires with low rolling resistance. Tires with low rolling resistance are characterized by lower fuel consumption in the car and thus result in lower emissions of carbon dioxide to the atmosphere [9,10]. Because of its favorable properties, BR is largely used not only in the tire industry (tire treads, inner tubes) but is also used in the production of tapes, V-belts, conveyor belts and drive belts [1,2]. Unfortunately, vulcanized BR reveals a poor wet skid resistance, low tear strength and tensile strength and the presence of double bonds in macromolecules makes butadiene rubber not resistant to aging factors, ozone and oil [11,12,13]. For this reason, BR is combined with other elastomers [14], e.g. with natural rubber [15,16], styrene-butadiene rubber [17,18] or chloroprene rubber (CR) [19,20].

Currently, elastomer blends prepared by mixing two or more elastomers are the subject of numerous scientific studies. The aim of production of elastomer blends is to reduce the production costs of rubber products and to improve the processability of elastomers. Unfortunately, there are few miscible elastomers due to too many factors that can determine it. These factors include composition, configuration, conformation, molecular weight, polydisperse, temperature and pressure of the process during the preparation of the blends. Thus, the rule is that polymers are incompatible due to positive mixing energy. As a result, mixing two elastomers creates a blend that is only miscible to a small extent. In general, polymers form multi-phase systems, thermodynamically incompatible, which is manifested by the presence of two glass transition temperatures. Such compounds have good properties only if the appropriate interactions between the ingredients are present [21,22,23].

Wang et al. found that the order of the ingredients added during the preparation of the elastomer blend has a significant influence on the compatibility of the elastomers in the BR/NR blend. As a compatibilizer in the butadiene/natural rubber (BR/NR = 30/70 by wt %) blend, a liquid butadiene-isoprene copolymer (LIR) was chosen. The best compatibility of the NR and BR in the NR/BR/LIR blend was obtained when the components were added in the following order: liquid butadiene-isoprene copolymer was first mixed with NR, and then blended with BR. The increased elastomers compatibility was manifested by a significantly reduced domain size of the dispersed BR phases [24].

BR also is thermodynamically immiscible with SBR. Wu et al. reported that surface-modified BR/SBR by silica particles enhanced the compatibility between SBR and BR. Additionally, the mechanical and thermal properties of SBR/BR blend had markedly improved by adding a coupling agent [25].

Whereas, Yao et al. studied the phase structure and the crystallization behavior of polymer blend containing butadiene rubber and polyethylene (PE). It has been found that both the phase structure and the crystalline morphology of PE depended on the composition and preparation of the sample. In the case of BR/PE blend with content of PE less than 10 phr, round-shaped PE microdomains were separately dispersed in the BR matrix. Whereas when the PE content was over 10 phr, some of the PE domains fused together and created randomly oriented lamellar aggregates [10,26].

The thermal and gamma irradiation resistance of composites based on waste rubber powder (WPR) filled butadiene rubber and chlorosulfonated polyethylene (BR/CSM) blends were investigated by Marković. The values of scorch time, vulcanization time and curing rate of these blends increased with increasing CSM content. In addition, the best mechanical properties (tensile strength, modulus at 100% elongation and hardness) were obtained for filled waste rubber powder vulcanizate containing 50 phr CSM and 50 phr BR. The improvement of these parameters proves the occurrence of synergism between the examined elastomers. Partial compatibility may be due to the ability of these elastomers to crystallize upon stretching [21].

Rubber blends containing butadiene rubber and chloroprene rubber (CR) are also known. These elastomers are different in polarization and form a typically thermodynamically immiscible system. To enhance the compatibility of BR and CR, styrene-butadiene-styrene copolymers (SBS) can be used as a compatibilizer. The addition of this substance to the BR/CR blend led to the formation of a homogeneous dispersion and an improvement of mechanical properties of the obtained vulcanizates [20].

The aim of the research was to evaluate the effectiveness of zinc as a new cross-linking substance with the simultaneous production of materials with good mechanical properties and a reduced fire hazard. In this investigation, butadiene rubber (BR) was chosen as the basic elastomer matrix, because of its low cost, excellent elasticity and other unique properties. Chloroprene rubber—after its cross-linking—has good tensile strength, weather resistance, heat resistance up to 100 °C, as well as better resistance to ozone, aging and non-polar solvents. The presence of the halogen element in the CR macromolecules makes CR highly resistant to fire. These properties resulted in the choice of CR as the elastomer matrix. In this study, the effect of the proportion of rubber in the blends and the amount of the cross-linking agent used on the useful properties of the resulting vulcanizates were analyzed. Additionally, mechanical properties, flammability and fire hazard were tested for the composites filled with silica, kaolin, chalk or montmorillonite.

## 2. Materials and Methods

### 2.1. Materials

Butadiene rubber (BR), SYNTECA^®^44 (*cis*-1.4 content: 97%, Mooney viscosity ML(1 + 4) at 100 °C: 39) was procured from Synthos S.A., Oswiecim, Poland. Chloroprene rubber (CR), BAYPREN^®^216 (bound chlorine content: 40%, Mooney viscosity ML(1 + 4) at 100 °C: 49 ± 5) was procured from LANXESS GmbH, Köln, Germany. Zinc (Zn), with a density of 7.13 g/cm^3^, procured from Aldrich Chemical Co., Steinheim am Albuch, Germany), was used as a cross-linking agent. Stearic acid, with a density of 0.94 g/cm^3^ from POCH S.A., Gliwice, Poland, was used as a dispersant. The following fillers were used in the work:

- Silica (Arsil), with a density of 0.15 g/cm^3^, from Rudniki S.A., Rudniki, Poland,
- Kaolin, with a density of 2.60 g/cm^3^ and bulk density of 0.47 g/cm^3^, from POCH S.A., Gliwice, Poland,
- Chalk, with a density of 2.71 g/cm^3^, from POCH S.A., Gliwice, Poland,
- Montmorillonite, NanoBent (MMT), modified with 4-order ammonium salt-containing hydroxyl groups (average grain size: 20–60 μm (81%), ≤20 μm (19%), layer spacing: 3.8–3.9 nm) from ZGM Zebiec S.A., Starachowice, Poland.

### 2.2. Preparation of Rubber Compounds

The CR/BR/Zn blends were made by means of a laboratory two-roll mill (roll sizes: 100 × 200 mm, roll temperature: 20–25 °C, friction ratio of 1:1.25). The mixing time was 6–10 min until all the components were thoroughly blended. First, both rubber (CR and BR) were mixed. The ingredients were added as follows: rubbers, dispersant, fillers and curing agent. Vulcanization time and temperature were determined based on rheometric tests carried out according to the standard ISO 3417:2008 [27] using a WG-02 oscillating disc vulcameter (disc amplitude: 2°, oscillation frequency: 1.7 ± 0.1 Hz). Torque was determined in a function of time upon heating at 160 °C.

The rubber compounds were vulcanized in steel molds of various shapes, depending on the test method. The molds were placed in a hydraulic press between platens electrically heated to 160 °C. The vulcanization was carried out at a pressure of 150 bar for the time resulting from rheometric tests. Poly(tetrafluoroethylene) films were applied to prevent adhesion of the blends to the vulcanization molds.

### 2.3. Characterization of Rubber Compounds

#### 2.3.1. Cure Characteristics

Scorch time (*τ*_02_), optimum cure time (*τ*_90_), the time required for the torque to reach 90% of the maximum torque, minimal torque (M_min_) and torque increment after 30 or 40 min of heating (∆M_30_, ∆M_40_) of the rubber compounds were determined from the cure curves. Torque increment (∆M) is the difference between the torque after heating and minimal torque values on the cure trace of a rubber blend and is an indication of cross-linking density changes in the elastomer compound. The cure rate index (*CRI*), a measure of the rate of curing, was calculated using the Equation (1):

(1) $$CRI\;=\;\frac{100}{\tau_{90}-\;\tau_{02}}$$

#### 2.3.2. Resistance to Swelling

Equilibrium swelling was performed in two solvents (toluene or heptane). Four samples (weight: 25–50 mg) cut from each vulcanizate were used for measurement. The samples were immersed in a solvent in a thermostatic chamber at 293 K for 72 h according to ASTM D 471 [28]. The surfaces of the samples swollen to equilibrium were washed with diethyl ether, weighed and then dried to constant weight at 50 °C and after 24 h were reweighed. Equilibrium volume swelling (*Q_v_*) was calculated from the Equation (2):

(2) $$Q_{v}=Q_{w}\cdot \frac{d_{v}}{d_{s}}$$

where: *d_v_* is the rubber density in the vulcanizate (g/cm^3^), *d*_s_ is the solvent density (g/cm^3^), and *Q_w_* is the equilibrium weight swelling (–) calculated from Equation (3):

(3) $$Q_{w}=\frac{m_{sw}-m_{d}}{m_{d}{}^{*}}$$

where: *m_sw_* is the weight of the swollen sample (g), *m_d_* is the weight of the dried sample (g), and m_d_* is the weight of the dried sample with mineral substances (g).

The cross-linking degree (*α_c_*) was determined from the Equation (4):

(4) $$\alpha_{c}=\frac{1}{Q_{v}}$$

#### 2.3.3. Extraction in Acetone

The real extract (*E_R_*) was calculated according to the Equation (5):

(5) $$E_{R}=\;\frac{m_{1}-m_{2}}{m_{1}},$$

where: *m*_1_ is the sample weight before extraction (mg), m_2_ is the sample weight after extraction (mg).

#### 2.3.4. Mooney–Rivlin Elasticity Constants

The Mooney–Rivlin parameters (the first (2C_1_) and second (2C_2_) elasticity constant) were calculated based on the Mooney–Rivlin Equation (6):

(6) $$2C_{1}+\lambda^{-1}\cdot 2C_{2}\;=\frac{P}{2A_{0}*\left(\lambda \;-{\;\lambda }^{-2}\right)}$$

where: P is the deformation force at λ (kG), λ is the deformation (λ = l/l_0_), l is the measuring section of the sample loaded with P (cm], l_0_ is the measuring section of the unloaded sample (cm), A_0_ is the cross-sectional area of the unloaded sample (cm^2^), 2C_1_ is the first elasticity constant (kG/cm^2^), and 2C_2_ is the second elasticity constant (kG/cm^2^).

#### 2.3.5. Mechanical Test

Mechanical properties of CR/BR/Zn composites (stress at 100% strain: S_e100_, stress at 200% strain: S_e200_, stress at 300% strain: S_e300_, tensile strength: TS_b_, elongation at break: E_b_) were determined using the a ZWICK 1435 universal machine (Germany) according to ISO 37:2007 [29]. Each determination was performed for five samples which were paddle-shaped (W2 type), and the width of the measured section was 4 mm. A constant speed of 500 mm/min was used.

#### 2.3.6. Payne Effect

The Payne effect determining the dynamic analysis of the vulcanizates produced was carried out using an ARES rheometer (plate-plate system, plate diameter: 20 mm, gap: 2 mm). The shear testing parameters were as follows: temperature: 25 °C, sample deformation rate: 10 rad/s, stress: from 0.1% to 150%, test force: 10 N. The Payne effect was performed for filled vulcanizates and compared with the reference sample. The Payne effect values (Δ*G*’) for composites were calculated from the Equation (7):

∆*G*’ = *G*’_min_(lim 10^−1^) − *G*’_max_(∞) (7)

where: *G*’_max_(lim10^−1^) is the storage modulus determined under the deformation of 10^−1^%, *G*’_max_(∞) is the storage modulus determined under the maximum deformation.

#### 2.3.7. Thermo-Oxidative Aging

The thermal aging of unfilled and filled CR/BR/Zn vulcanizates was carried out in a forced circulating aging oven at 70 °C for 7 days. After conditioning at room temperature for 24 h, the changes of mechanical properties (stress at 100%, 200% or 300% strain, tensile strength, elongation at break) were evaluated based on the aging coefficient (K) based on the Equation (8):

(8) $$K\;=\;\frac{{\mathrm{TS}}_{b}^{\prime }\cdot E_{b}^{\prime }}{{\mathrm{TS}}_{b}\cdot E_{b}}$$

where: TS’_b_ is the tensile strength after thermo-oxidative aging (MPa), TS_b_ is the tensile strength before thermo-oxidative aging (MPa), E’_b_ is the elongation at break after thermo-oxidative aging (%), and E_b_ is the elongation at break before thermo-oxidative aging (%).

#### 2.3.8. Flammability Tests

The flammability of the vulcanizates was determined by the oxygen index method and the time of burning in air [30]. Samples were prepared in the form of cuboid with dimensions: 50 × 10 × 4 mm. The nitrogen flow was constant and amounted to 400 L/h, while the oxygen concentration was chosen so that the sample was completely burned in 180 ± 10 s. The tip of the sample was ignited for 5 s with a gas burner supplied with a gas mixture (propane—butane). The oxygen index (OI) was calculated as a percentage of the volume of oxygen and nitrogen in the mixture according to the Equation (9):

(9) $$\mathrm{OI}\;=\frac{O_{2}}{O_{2}+N_{2}}\cdot 100\%$$

where: O_2_ is the oxygen flow rate [L/h], and N_2_ is the nitrogen flow rate [L/h].

The time of burning in air (t_c_) was determined using the same samples as for the oxygen index. The vertically positioned samples were ignited for 5 s using a gas burner. After this time, the following parameters were measured: the burning time of the sample or the time after which the sample was extinguished. The measurement was repeated five times.

A cone calorimeter built by Fire Testing Technology was used to measure ignition characteristic, heat release rate, and sample mass loss rate according to ISO 5660-1. An external radiant heat flux of 35 kW/m^2^ was applied. All the samples were measured in the horizontal position and wrapped with thin aluminum foil except for the irradiated sample surface. The standard uncertainly of the measured heat release rate was ± 10%. Based on the results the fire hazard was calculated as the fire propagation rate (1/t_flashover_) from the Equation (10):

(10) $$\frac{1}{t_{\mathrm{flashover}}}\;=\frac{{\mathrm{HRR}}_{\max}}{\mathrm{TTI}}$$

## 3. Results and Discussion

### 3.1. Effect of Elastomers Mass Ratio on Cross-Linking and Properties of CR/BR/Zn Blends

#### 3.1.1. Cure Characteristic of CR/BR/Zn Blends

The main purpose of cross-linking is to create cross-links between the elastomer macromolecules, which leads to the creation of a three-dimensional network. Many systems have been developed for the vulcanization of rubbers, therefore a cross-linking process can be carried out according to the different mechanisms and in the presence of different curing agent such as sulfur, organic peroxides, metal oxides, phenolic resins. In the first stage of the work, the influence of the mass ratio of elastomers in the zinc cross-linked CR/BR blends on the kinetics and selected properties of these blends was investigated. This made it possible to select the appropriate compositions for further stages of research, because the selection of proper elastomers proportion for the blends mainly determines the properties of the products obtained.

The cure characteristics: scorch time (τ_02_), cure time (τ_90_), minimal torque (M_min_), torque increment after 30 or 40 min of heating (∆M_30_, ∆M_40_) for CR/BR blends, are shown in Table 1. These parameters were dependent mainly on the proportion of the elastomers in the prepared CR/BR blends. From the cure characteristic of the composites, it can be observed that the shortest scorch time (τ_02_ = 1.97 min) was obtained for the sample containing 75 parts of chloroprene rubber and 25 parts of butadiene rubber, whereas the CR/BR (20/80 by wt %) blend was characterized by the longest scorch time (τ_02_ = 5.37 min). The cure time (τ_90_) decreased with an increase in the BR content. For example, the τ_90_ value for pure CR was 53.55 min while for the CR/BR blend (40/60 by wt %) was only 8.08 min. The minimal vulcametric torque (M_min_), which is a measure of the viscosity, reached similar values for compositions with 20 and 25 parts of butadiene rubber. The greater amount of butadiene rubber in the CR/BR blends led to a marked increase in viscosity, which is closely related to the dilution effect because pure BR shows higher viscosity compared to the viscosity of CR. The cross-linking degree in this study was determined by the torque increment after 30 or 40 min of heating. The greater the torque increment, the higher cross-linking density. The blends containing from 100 to 75 parts of chloroprene rubber can be effectively cross-linked with zinc, while the incorporation of more parts of BR into the CR/BR blends resulted in a decrease in the cross-linking degree. The butadiene rubber is not susceptible to the curing in the presence of zinc, which was confirmed by the lack of the torque increment after the heating. No susceptibility of BR to the cross-linking in the presence of Zn leads to the decrease in the cross-linking degree for the CR/BR blends containing more than 25 parts of BR (Table 1).

Surprisingly, the speed of vulcanization increased with the decrease in CR amount in the blends, which is evident from the significant increase in the cure rate index. Such a high value of CRI for the CR/BR (20/80 by wt %) blend is most likely due to the predominant amount of butadiene rubber in the composition, which is not cross-linked with zinc. Therefore, a small amount of chloroprene rubber in the blend is rapidly cross-linked in the presence of zinc, with a slight increase in torque increment after 30 or 40 min of heating. Based on vulcametric studies, the temperature of 160 °C was selected as the curing temperature for all CR/BR/Zn blends. The vulcanization time for all composites was 30 min.

The results obtained through vulcametric studies were confirmed by the swelling degree in heptane or toluene (Table 2), which provides information on the network chain density (determined by the swelling ratio) and the percentage of the soluble fraction (i.e., the percentage of chains that do not belong to networks). In the case of cross-linked elastomers, the swelling is limited due to the generation of cross-links connecting the polymer chains, which prevents their extension and relaxation [22]. The swelling data show that the composite containing only CR is characterized by higher solvent resistance than the CR/BR vulcanizate, as evidenced by the lowest Q_v_^H^ = 0.43 mL/mL in heptane and Q_v_^T^ = 5.39 mL/mL in toluene. The presence of BR in vulcanizates leads to an increase in the swelling equilibrium state in both heptane and toluene. The Q_v_^T^ value in toluene for the CR/BR (20/80 by wt %) compound exceeded 23 mL/mL. Consequently, the reduction in the degree of cross-linking increases the penetration of the solvent into the cured CR/BR/Zn composites. The conducted tests clearly confirmed the impossibility of cross-linking of butadiene rubber with zinc, as evidenced by the total solubility of such a sample in toluene.

The similar correlation in the cross-linking degree was observed in the determined Mooney–Rivlin elasticity constants. The values of the first elasticity constant (2C_1_) are directly proportional to the cross-linking density. The sample containing only butadiene rubber and vulcanizates containing 20 weight parts of chloroprene rubber and 80 weight parts of butadiene rubber have not been tested for elasticity constant because samples would be destroyed during the test, which indicates no curing. For the CR/BR (80/20 by wt %) vulcanizate, the value of 2C_1_ was 1.53 kG/cm^2^ (Table 2). The highest value of first elasticity constant was obtained for the CR vulcanizate (2C_1_ = 1.94 kG/cm^2^). The 2C_1_ parameter can be correlated with equilibrium swelling values—the higher the constant value, the lower the swelling of the vulcanizate. This relationship is observed in the examined CR/BR/Zn products. The value of the second elasticity constant (2C_2_) can be interpreted as a deviation from the ideal elasticity of the rubber. The highest value of 2C_2_ (2.81 kG/cm^2^) was obtained for the CR/BR (75/25 by wt %) vulcanizate, whereas for the CR/BR (40/60 by wt %) vulcanizate, 2C_2_ = 0.42 kG/cm^2^. The calculated Mooney–Rivlin elasticity constants indicate that the composition of the CR/BR/Zn blend clearly determines the degree of cross-linking of the vulcanizates. The use of more amount of chloroprene rubber in the blend is advantageous because it guarantees the formation of more cross-links, which confirms the greater degree of cross-linking. Whereas, the increase in the content of butadiene rubber in the blend increases the plasticity of vulcanizates.

The vulcanizates were extracted in boiling acetone in order to determine the content of non-rubber components in the blends of chloroprene and butadiene rubber. It was found that the least extracted substances occurred for the vulcanizate containing only CR and for the CR/BR (80/20 by wt %) vulcanizate, for which E_R_ ~0.086 mg/mg (Table 2). This demonstrates that a significant cross-linking degree was achieved in these vulcanizates. The lower amount of CR in the samples leads to a higher amount of real extract which may indicate a smaller extent of cross-linking of such blends. These results correlate with the calculated cross-linking degree (α_c_) (Table 2).

#### 3.1.2. Mechanical Properties of the CR/BR/Zn Vulcanizates

**Figure 1:**
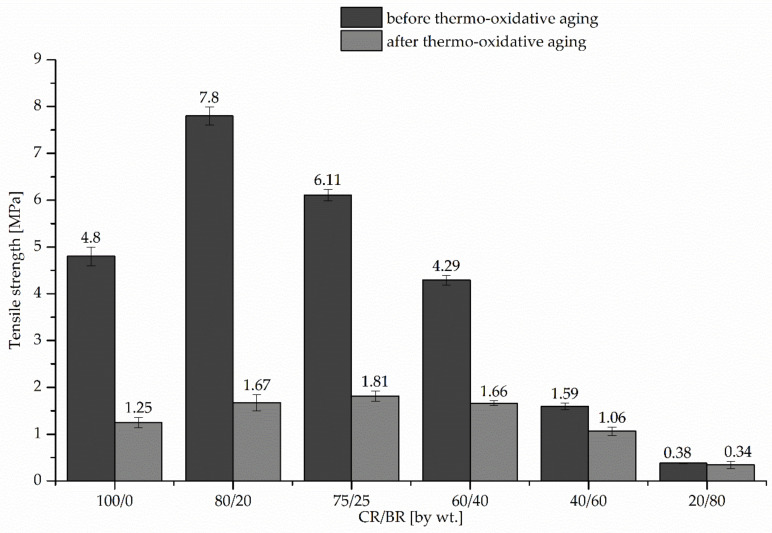
Effect of the blend ratio (CR/BR) on mechanical properties before and after thermo-oxidative aging.

The CR/BR/Zn vulcanizates were tested for: stress at 100%, 200% or 300% strain, tensile strength, elongation at break and resistance to thermo-oxidative. Test results for the investigated mechanical properties of CR/BR/Zn products are presented in Table 3 and Figure 1. The amount of butadiene rubber used to produce CR/BR/Zn vulcanizates affects the parameters such as the stress at 100%, 200% or 300% strain, the tensile strength and the elongation at break. The values of S_e100_, S_e200_, S_e300_ decreased with the increasing amount of BR in CR/BR sample. The stress at 100% strain changed from 0.60 MPa for the CR/BR (80/20 by wt %) vulcanizate to 0.20 MPa for the CR/BR (20/80 by wt %) sample, which indicates the higher the content of CR the higher the stiffness of tested compounds. The tensile strength depends on the nature and type of cross-links, cross-link density and chemical structure of the used elastomers. In the case of the sample with CR only, tensile strength was 4.80 MPa, whereas the composite containing 20 or 25 parts of BR was characterized by a much higher value of this parameter (respectively, 7.80 and 6.11 MPa). Increased TS_b_ value may be associated with satisfying compatibility between CR and BR or the formation of bonds between the macromolecules of tested elastomers [31,32,33]. Incorporation of at least 40 parts of BR into CR led also to the dilution effect of the system, which resulted in a decrease in tensile strength. The smallest elongation at break (E_b_ = 495%) was measured for the CR cured with zinc, while the presence of butadiene rubber in the tested compounds resulted in a significant increase in this parameter. For the CR/BR (20/80 by wt %) vulcanizate the E_b_ value exceeded 1000%.

Based on the conducted aging tests (Table 3, Figure 1), it was found that CR/BR composites cross-linked with zinc did not show satisfactory resistance to thermo-oxidative factors. All samples had a very small tensile strength, and for most of them, short elongation at break was observed. The highest TS’_b_ value (1.81 MPa) was obtained for the CR/BR (75/25 by wt %) vulcanizate, whereas the smallest value of tensile strength (TS’_b_ = 0.34 MPa) was achieved for the sample containing 20 parts of chloroprene rubber. The use of zinc as a cross-linking substance significantly reduces the resistance to thermo-oxidative aging not only for CR/BR vulcanizates but also for the sample containing only CR (K = 0.115), which vulcanizates are widely recognized as resistant to aging. The high aging coefficient for the CR/BR (20/80 by wt %) vulcanizate is due to the slight difference in small tensile strength before and after thermo-oxidative aging.

### 3.2. Effect of Zinc Amount on Cross-Linking and Properties of CR/BR Blend

#### 3.2.1. Cure Characteristic of CR/BR Blend with Different Amount of Zn

In the second stage of the work, it was decided to investigate whether the amount of zinc influences the cross-linking of the CR/BR blends and the properties of vulcanizates produced. Therefore, the CR/BR (80/20 by wt %) blends containing 1, 2, 3, 4 or 5 phr of Zn were prepared (Table 4).

**Figure 2:**
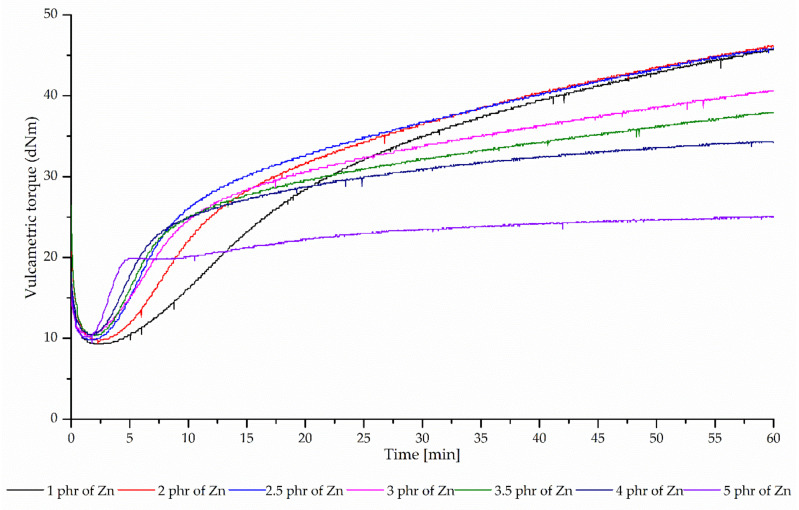
Vulcametric torque as a function of the cure time for the CR/BR (80/20 by wt %) blends cross-linked with different amount of zinc at 160 °C.

The data show that even a small amount of zinc causes effective cross-linking of CR/BR (80/20 by wt %) blends (Figure 2). The incorporation of 1 phr of Zn results in a scorch time of 4.82 min. However, the incorporation of 5 phr of Zn causes the shortening of τ_02_ value to 1.78 min. The amount of curing agent does not affect the minimal torque which was ~9.8 dNm for all samples. Based on the torque increment after 30 min of heating (ΔM_30_) it was found that the most effective cross-linking of the CR/BR blend occurs when the zinc content does not exceed 2.5 phr. The incorporation of more amount of Zn into the blend resulted in a reduction in the cross-linking degree. The highest value of ΔM_30_ (27.3 dNm) was noted for the blend cured with 2.5 phr of Zn, while the lowest value ΔM_30_ (14.0 dNm) was found for the sample containing 5 phr of Zn. It was noticed that the speed of vulcanization increased with the increase in zinc amount, which is evident from the significant increase in the cure rate index. The CRI value for the CR/BR blend cured with 1 phr of zinc was 2.51 min^−1^, but this parameter for the blend with the highest amount of Zn equal 4.92 min^−1^.

This relationship was also observed for the determination of equilibrium volume swelling in heptane and toluene and Mooney–Rivlin elasticity constants. For the CR/BR/Zn (80/20/2.5 by wt %) vulcanizate the Q_v_^T^ value was 7.48 mL/mL, while for the composite cross-linked with 5 phr of zinc the highest Q_v_^T^ value (10.34 mL/mL) was observed. It indicates the smallest cross-linking degree for this sample. However, the α_c_ value, which determines the cross-linking degree, was similar for all vulcanizates and equal 0.10. In addition, the highest value of the first elasticity constant (2C_1_~1.4 kG/cm^2^) was achieved for vulcanizates containing 2–4 phr of zinc. Whereas, in the case of the CR/BR/Zn (80/20/5 by wt %) composite the lowest 2C_1_ value (1.13 kG/cm^2^) and the highest 2C_2_ value (7.44 kG/cm^2^) were noticed. Whereas, the smallest 2C_2_ value (1.47 kG/cm^2^) was obtained for the vulcanizate containing 2.5 phr of Zn. The measurements of extraction with boiling acetone (Table 4) showed that as the zinc amount in the vulcanizate increased, the value of the real extract (E_R_) also increased. The lowest values of E_R_ (~0.085 mg/mg) were recorded for vulcanizates in which the zinc content did not exceed 2.5 phr. Whereas the highest values of real extract (~0.122 mg/mg) were obtained for the blend cross-linked with 4 or 5 phr of zinc. These results confirm that too much zinc as a cross-linking substance leads to a decrease in the cross-linking degree, which confirms the previously described results from vulcametric tests. Then the network created during the cross-linking has a small number of bonds. The use of at least 2 phr of Zn causes the creation of a network with a significant number of bonds. To obtain a CR/BR vulcanizate with a correspondingly high amount of cross-links, zinc in an amount of 2 to 4 phr must be used.

#### 3.2.2. Mechanical Properties of CR/BR Vulcanizates with Different Amount of Zn

It was also established that the amount of zinc affects the properties of the obtained rubber products. The vulcanizate (CR/BR/Zn = 80/20/5 by wt %) with the lowest cross-linking degree had also the lowest tensile strength (TS_b_ < 5 MPa). Whereas the best mechanical properties were obtained for the sample cross-linked with 2.5 phr of zinc. In this case, the tensile strength was increased by 22% (TS_b_ = 8.60 MPa). Additionally, it was found that the zinc amount did not significantly affect stress at 100%, 200% or 300% strain. Elongation at break had the lowest value (456%) for the CR/BR compound cured with 1 phr of Zn. The highest value of E_b_ was observed for the CR/BR compound cured with 4 phr of Zn (Table 5). For comparison, the chloroprene rubber conventionally cross-linked (e.g., with 4 phr of MgO and 5 phr of ZnO) achieves the tensile strength at break of 7.14 MPa [34]. However, for the butadiene rubber cross-linked with 1.3 phr of sulfur in the presence of 1.3 phr of N-cyclohexyl-2-benzothiazolesulfenamide (CBS), the tensile strength does not exceed 1.2 MPa, and elongation at break is 120% [18]. It is worth noting that the standard cross-linked CR or BR is characterized by worse mechanical properties than the CR/BR/Zn vulcanizates proposed in this paper. However, too high amount of zinc (over 5 phr) used to cross-link the CR/BR composites is not recommended, as it causes the deterioration of the performance properties of the vulcanizates resulting from a lesser cross-linking degree.

It has been found that the use of zinc in an amount of 2.5 to 4 phr leads to the formation of a network with a large number of cross-links, which provides compounds with optimal mechanical properties. An increase in the amount of zinc above 4 phr deteriorates the properties of the vulcanizates. This may indicate that only a certain amount of zinc reacts with tested rubbers forming the bonds. The most optimal is the use of 2.5 phr of Zn as the cross-linking agent. Using smaller amount of cross-linking agent also has an economic argument as it leads to less expensive final products.

Unfortunately, produced elastomer materials were characterized by low resistance to thermo-oxidative aging, because samples exposed to aging factors showed significant deterioration of tensile properties. In the case of the CR/BR/Zn (80/20/2.5 by wt %) compound the tensile strength decreased from 8.60 MPa to 1.39 MPa, while the elongation at break decreased from 750% to only 29%. An aging coefficient of the obtained vulcanizates did not exceed 0.04 (Table 5).

### 3.3. Mechanical, Dynamical and Thermal Properties of Filled CR/BR/Zn Vulcanizates

Different types of additives are used in the processing of rubbers into products include vulcanization accelerators, activators, plasticizers, dyes, pigments and fillers. Fillers are solid substances capable of changing the physical and chemical properties of rubber products by surface interaction or its lack thereof and by their own physical characteristics. Using a filler into the elastomers matrix allows to obtain the rubber composites of useful features. The fillers modify the physical and, to some extent, the chemical properties of the vulcanizates [21]. In this study, silica, kaolin, chalk (in the amount of 30 phr) and montmorillonite (MMT, in the amount of 5 phr) were used as fillers into CR/BR/Zn (80/20/2.5 by wt %) compounds. Based on the obtained results, it was found that the incorporation of the filler into the studied blend significantly affects the cross-linking course and on the selected properties of cured products (Table 6).

The cross-linking degree for the sample filled with kaolin or chalk was slightly lower compared to the unfilled CR/BR/Zn blend. The torque increment after 40 min of heating for the unfilled composite was 30.3 dNm, whereas ∆M_40_ for the CR/BR/kaolin or CR/BR/chalk blend was 24.2 dNm or 29.4 dNm, respectively. More than twice the lower value of this parameter was observed in the case of the blend filled with silica (∆M_40_ = 13.3 dNm). The decrease in this parameter is due to the fact that the precipitated silica adsorbs curative molecules on their surfaces, rendering the deactivation of vulcanization, and thus lower the cross-linking density of the prepared compounds is observed [35,36]. The viscosity of the CR/BR/Zn blend clearly increased in the sample containing silica, as evidenced by the highest value of the minimal moment (M_min_ = 21.2 dNm). This marked increase in viscosity is due to the flow restriction effect caused by solid particles of silica [35]. A similar value of minimal torque (M_min_~11.4 dNm) for the blend filled with kaolin, chalk or MMT was observed. In the case of the CR/BR/Zn blends filled with silica, kaolin or chalk the speed of vulcanization was similar (CRI~2.74 min^−1^). However, for the MMT-filled blend the CRI value equal 3.25 min^−1^, which may be due to the small filler’s amount in the total composition (only 5 phr). The lowest equilibrium swelling value (Q_V_^T^ = 6.52 mL/mL) in toluene was achieved in the case of chalk-filled vulcanizates, which is probably related to the strong adhesion of this filler to the polymer, which cannot be removed by the solvent molecules [36]. However, the highest equilibrium swelling value in toluene (Q_V_^T^ = 11.56 mL/mL) was obtained for vulcanized filled with MMT, which indicates the lowest resistance to organic solvents of this compound.

A significant improvement of the tensile strength (Table 6) is evident in the case of vulcanizate with the silica, kaolin or chalk relative to unfilled and MMT-filled CR/BR composites. The tensile strength for the filled (with silica, kaolin, chalk) CR/BR products was in the range from 9.62 to 10.08 MPa. The silica contains large amount of silanol groups (Si–OH) on the surface, it is considered as a highly polar and reactive filler. Therefore, silica is more compatible with chloroprene rubber. Hydrogen bonds are likely formed between CR and silica. It is possible that the positive hydrogen cation present in the silanol group of silica interacts with the negative chlorine atom in CR [35]. The creation of additional bonds between silica and CR contributes to the improvement of mechanical properties. Kaolin and chalk reveal some chemical affinity to different elastomer into which they are incorporated to improve the physico-mechanical parameters. On the unmodified chalk surface, the entanglement of polymer chains is observed. Additionally, the chalk surface may react also with stearic acid present in the system, which leads to decreases anisotropy of chalk particles and at the same time increases its specific surface area. Therefore, the incorporation of the chalk improves the strength parameters of CR/BR/Zn vulcanizates [37]. In the case of the CR/BR/Zn vulcanizates filled with kaolin, good mechanical properties can be caused by the particle’s shape of this filler that form thin lamellas, so that the tensile strength along the lamella direction is good [38]. Conventional fillers, optionally with a coupling agent, are usually processed by direct incorporation into the rubber mixes. The incorporation of nanofillers into an elastomer makes it more difficult to obtain the good dispersion. Therefore, the successful application of nanofillers depends mainly on a good dispersion of these substances [39]. The worst mechanical properties obtained for the vulcanizate filled with MMT may be related to incorrect dispersion of the filler in the elastomer matrix or the lowest cross-linking degree of this vulcanizate (∆M_40_ = 17.8 dNm, Q_V_^T^ = 11.56 mL/mL). Additionally, the most attractive feature of nanocomposite is achieving excellent reinforcement effect only by very small filler loadings [40]. The type of filler did not affect the value of elongation at break. For all samples, E_b_ was equal to about 660%. Nevertheless, the presence of silica and chalk in the vulcanizates produced increased their resistance to thermo-oxidative aging. In the case of vulcanizates containing silica, the tensile strength decreased from 10.08 MPa to 4.42 MPa, whereas TS’_b_ for the material filled with kaolin or montmorillonite was approximately 2.39 MPa. The low elongation at break that was examined after thermo-oxidative aging is also the reason for the very low aging coefficient (K ≤ 0.04).

**Figure 3:**
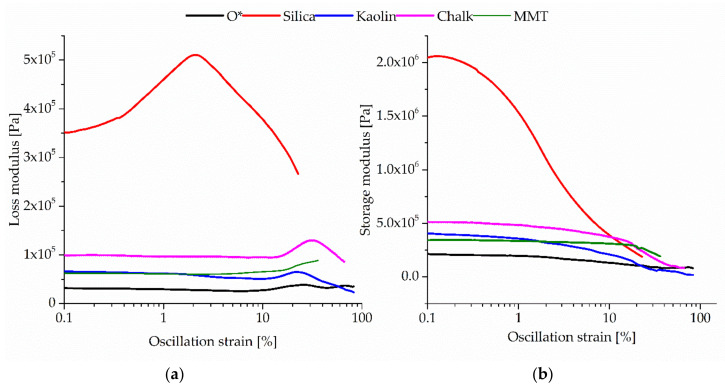
Storage modulus (**a**) and loss modulus (**b**) of unfilled and filled CR/BR/Zn vulcanizates.

These conclusions are consistent with the results of the dynamic analysis of tested materials. The calculated Payne effect allows to determine the interactions between the particles of fillers in the filled compounds. In this test, the dynamic properties were studied with strain sweep from a very small deformation to high deformation [41]. In the filled vulcanizates, the reduction of storage modulus (G′) and the increment of loss modulus (G″) with the increasing amplitude deformation were observed (Table 7, Figure 3). For comparison, the dynamic properties of the unfilled sample were also presented. The changes in the minimum and maximum modules in filled composites were observed. The biggest decrease in the storage modulus was noticed in the case of the CR/BR/Zn/silica, which may result from the creation of the structure by the fillers interactions. Additionally, the decrease in the modulus was due to the increase in the surface area. Other fillers as kaolin, chalk and montmorillonite were less formable to create the ‘structure’, which was manifested by a much lesser change modulus.

**Figure 4:**
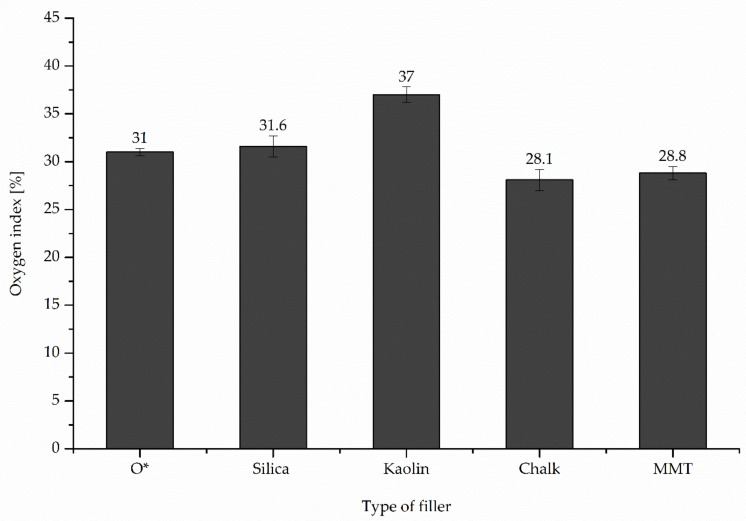
Oxygen index (OI) of unfilled and filled CR/BR/Zn vulcanizates.

Negative features of rubber materials include low thermal stability under exploitation conditions and too high flammability. Thermal properties of elastomers depend on the chemical structure of rubber macromolecules, method and degree of cross-linking and composition of rubber compounds. The conventionally cured butadiene rubber is a combustible material, determined by the oxygen index (OI) method. The OI value of conventional BR vulcanizates is equal to 17%. Chloroprene rubber cross-linked in a standard way, i.e., with zinc oxide and magnesium oxide, has a higher oxygen index (26%), which classifies it as a flame-retardant material. Based on this data thermal properties of the unfilled and filled CR/BR/Zn vulcanizates were investigated in this work. It was found that the combination of both elastomers and their cross-linking in the presence of zinc led to the production of incombustible materials. In the case of the CR/BR/Zn/kaolin, the highest value of the oxygen index (37%) was observed. Whereas the unfilled product or filled with silica, chalk or montmorillonite had the oxygen index above 28% (Figure 4).

During the pyrolysis process, the fillers (kaolin, chalk) are decomposed with the release of large amounts of inert gases, e.g., carbon dioxide, water vapor. These gases dilute the gaseous, flammable, thermal decomposition products pass to the flame as well as they limit diffusion of oxygen into the combustion zone, which reduces the rate of thermal decomposition of the burned materials and increases their fire resistance.

The possibility of producing strong polymer-filler interactions (in the case of silica) helps to reduce the mobility of the segmental macromolecules, which results in an increase in the thermal stability of the vulcanizate and a reduction in its flammability [42]. Very good fire properties of nanocomposites, such as the CR/BR/Zn/MMT compound, result from the structure of these materials. High temperatures and flames create a boundary layer on the surface of the material without cracks, isolating the polymer from fire and restricting access of combustible polymer decay products to the combustion zone [43,44]. Results obtained demonstrate the strong flame resistance of the tested CR/BR/Zn compositions, which does not require the incorporation of flame retardants.

The time of burning in air (t_c_) for four tested vulcanizates (unfilled and filled with silica, kaolin or MMT) did not exceed 5 s, which classifies these materials as self-extinguishing. The t_s_ value of the CR/BR/Zn/chalk vulcanizate was 12 s. Burned samples behave differently, depending on the blend composition. All vulcanizates burn with a green glow, more or less intense, which is the result of the burning of chlorine present in CR. The cured CR/BR/Zn compounds containing silica were burned with a flame, which covered the sample in layers. Additionally, during the combustion, the sparking occurred and the sample delaminated and turn to ash. Due to the presence of kaolin in the CR/BR/Zn vulcanizate, the sample retained its shape after the burning, and the surface was covered with a layer of ash. The chalk-filled vulcanizate retained its shape after the burning, and the surface was covered with a black coating. The CR/BR/Zn vulcanizate containing montmorillonite burned with a smoky flame and after testing a frayed skeleton of the sample remained.

**Figure 5:**
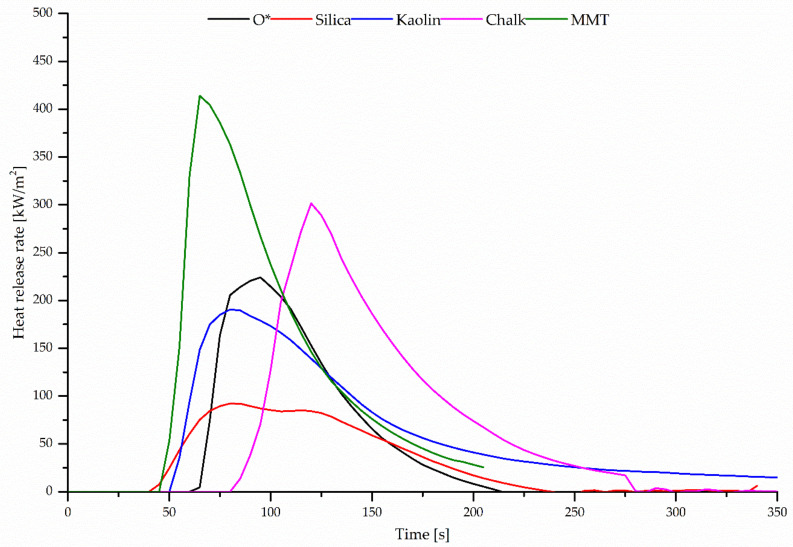
Heat release rate (HRR) versus incineration time of unfilled or filled CR/BR/Zn (80/20/2.5 by wt %) compounds.

Although the method of oxygen index is commonly used for testing the flammability of polymers, the OI values obtained can only be used for a comparative assessment of flammability. It should be taken into account that the OI method is strict laboratory test. More detailed information is provided by the cone calorimeter (real fire conditions). It is assumed that the basic fire hazard properties of the material include: ignitability measured as the time to sustained ignition (TTI), total heat release (THR), heat release rate (HRR), effective heat of combustion (EHC) and average specific mass lost rate (MLR). The analysis of data presented in Table 8 leads to the conclusion that the tested vulcanizates are materials with high resistance to burning. The average maximum heat release (HRR_max_) of the unfilled CR/BR/Zn vulcanizates equal only 224.23 kW/m^2^. For the CR/BR/Zn sample filled with silica, the HRR_max_ value decreased to 92.20 kW/m^2^. The presence of nanofillers (MMT) in the tested compounds resulted in a slightly higher this parameter (414.01 kW/m^2^) (Figure 5). It should be stressed that during the combustion of the tested materials, regardless of their composition, the heat release rate is very low as compared to the commonly used rubber products such as cured acrylonitrile–butadiene rubbers, NBR18 and NBR39, for which this parameter amounts to 3569.23 and 3115.28 kW/m^2^, respectively [45].

**Figure 6:**
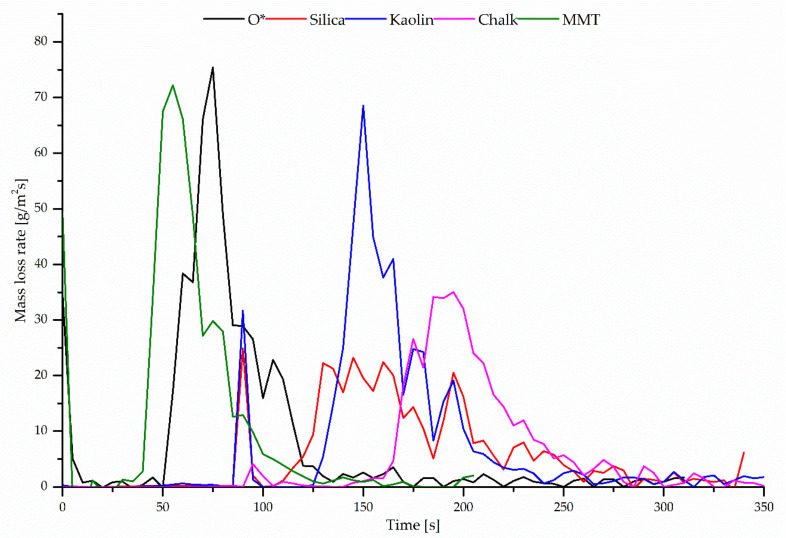
Mass loss rate (MLR) versus incineration time of unfilled or filled CR/BR/Zn (80/20/2.5 by wt %) compounds.

The mass loss rate (MLR) of the studied composites, which proves the dynamics of material combustion dynamics, reached the highest value (49.99 g/m^2^·s) in the case of the CR/BR/Zn vulcanizate containing montmorillonite. During the combustion of this compound, the greatest amount of heat is released, and the sample was burned in 97.7% (Figure 6).

Based on the results obtained with the cone calorimeter the fire hazard was calculated, which is connected with the fire propagation rate (1/t_flashover_), i.e., the inverse time to reach the flashover effect [46]. The lowest fire hazard was observed for the CR/BR/Zn vulcanizates filled with silica or chalk (1/t_flashover_ equal 3.29 and 3.97 kW/m^2^·s, respectively). Again, among the tested compounds, the sample containing nanofiller was characterized by the highest 1/t_flashover_ value (9.63 kW/m^2^·s). It is worth noting that 1/t_flashover_ assessed on the basis of the ignition time and HRR_max_ of all the vulcanizates produced is much smaller (Table 8) as compared with cured acrylonitrile-butadiene rubbers, NBR18 and NBR39, whose corresponding values amount to 65.87 and 65.88 kW/m^2^·s, respectively [45]. The obtained test results clearly indicate that the manufactured CR/BR/Zn products, both unfilled and filled, are non-flammable and pose a low fire hazard.

## 4. Conclusions

Elastomer composites containing chloroprene and butadiene rubbers (CR/BR) can be effectively cross-linked with zinc, and the obtained cross-linking density obtained largely depends on the composition of the blend, including the mass ratio of both elastomers and the amount of zinc used. The best processing and performance properties of CR/BR blends can be obtained if the amount of chloroprene rubber is not less than 60 phr and the zinc’s content is not more than 2.5 phr. The results revealed that the cross-linking degree decreased with the increasing amount of butadiene rubber in the blends. The mechanical properties of cured CR/BR composites, based on stress at strain and tensile strength, depended on both the proportion of elastomers in composites, the zinc’s amount, as well as the presence and type of filler. The tensile strength for the filled CR/BR/Zn compounds was about 10 MPa. The presence of the filler affects both the cross-linking and the properties of the obtained vulcanizates. The incorporation of chalk into the elastomer matrix increased the degree of cross-linking of the CR/BR/Zn blend. The best strength properties were demonstrated by vulcanizates containing 30 parts of chalk, kaolin or silica. The flammability of CR/BR/Zn vulcanizates has been investigated before and after the filling. The parameters assessed by the oxygen index method and cone calorimetry, characterizing the behavior of the tested CR/BR/Zn vulcanizates under fire conditions, have shown that they constitute a low fire hazard and can be considered as non-flammable materials. This is confirmed by parameters such as the heat release rate effective heat of combustion and fire propagation rate.

## 5. Patents

Polish Patent No. 234,661 (from 2020) was created based on the results from the work reported in this manuscript. The title of the patent is “Method of cross-linking and modification of elastomer blends”.

## Figures and Tables

**Table 1:** The cure characteristic of the chloroprene rubber/butadiene rubber (CR/BR) blends cross-linked with zinc (3 phr of Zn) at 160 °C.

CR/BR (Blend Ratio)	τ_02_ (min)	τ_90_ (min)	M_min_ (dNm)	∆M_30_ (dNm)	∆M_40_ (dNm)	CRI (min^−1^)
100/0	3.10	53.55	10.3	26.0	34.1	1.98
80/20	2.78	40.57	10.4	23.3	25.9	2.65
75/25	1.97	38.08	10.2	22.0	24.0	2.77
60/40	3.13	28.42	12.3	15.9	17.0	3.95
40/60	2.93	8.08	14.5	10.7	10.7	19.42
20/80	5.37	9.65	17.2	5.6	6.1	23.36
0/100	-	-	24.4	-	-	-

τ_02_—scorch time, τ_90_—cure time, M_min_—minimal torque, ∆M_30_, ∆M_40_—torque increment after 30 or 40 min of heating, CRI—cure rate index.

**Table 2:** The cure characteristic of the CR/BR blends cross-linked with zinc (3 phr of Zn) at 160 °C for 30 min.

CR/BR (Blend Ratio)	Q_v_^T^ (mL/mL)	Q_v_^H^ (mL/mL)	α_c_ (–)	2C_1_ (kG/cm^2^)	2C_2_ (kG/cm^2^)	E_R_ (mg/mg)
100/0	5.39	0.43	0.19	1.94	2.74	0.083
80/20	10.14	1.27	0.10	1.53	2.25	0.088
75/25	11.10	1.62	0.09	1.56	2.81	0.141
60/40	14.14	2.60	0.07	1.23	2.56	0.169
40/60	14.81	3.32	0.07	0.57	0.42	0.274
20/80	23.06	5.38	0.04	-	-	0.364
0/100	D	D	-	-	-	D

Q_v_^T^, Q_v_^H^—equilibrium swelling degree in toluene or heptane, α_c_—cross-linking degree in toluene, 2C_1_, 2C_2_—first and second Mooney–Rivlin elasticity constant, D—dissolvable.

**Table 3:** Effect of the blend ratio (CR/BR) on mechanical properties of vulcanizates.

CR/BR (Blends Ratio)	S_e100_ (MPa)	S_e200_ (MPa)	S_e300_ (MPa)	TS_b_ (MPa)	E_b_ (%)	S’_e100_ (MPa)	TS’_b_ (MPa)	E’_b_ (%)	K (-)
100/0	0.94	1.56	2.31	4.80	495	0.77	1.25	218	0.115
80/20	0.60	0.88	1.19	7.80	796	-	1.67	60	0.016
75/25	0.65	1.04	1.50	6.11	665	-	1.81	49	0.022
60/40	0.49	0.74	1.01	4.29	727	-	1.66	32	0.017
40/60	0.34	0.47	0.58	1.59	738	0.42	1.06	426	0.385
20/80	0.20	0.21	0.24	0.38	1075	0.16	0.34	999	0.831

S_e100_, S_e200_, S_e300_—stress at 100%, 200%, 300% strain, TS_b_—tensile strength, E_b_—elongation at break, S’_e100_—stress at 100% strain after thermo-oxidative aging, TS’_b_—tensile strength after thermo-oxidative aging, E’_b_—elongation at break after thermo-oxidative aging, K—aging coefficient.

**Table 4:** The cure characteristic of the CR/BR (80/20 by wt %) blends cross-linked with different amount of zinc at 160 °C.

Zn (phr)	τ_02_ (min)	τ_90_ (min)	M_min_ (dNm)	∆M_30_ (dNm)	∆M_40_ (dNm)	CRI (min^−1^)	Q_v_^T^ (mL/mL)	Q_v_^H^ (mL/mL)	2C_1_ (kG/cm^2^)	2C_2_ (kG/cm^2^)	E_R_ (mg/mg)
1	4.82	44.60	9.3	25.7	30.1	2.51	8.15	1.07	1.24	1.87	0.084
2	3.68	43.90	9.4	27.0	31.0	2.49	7.67	1.12	1.34	2.07	0.085
2.5	3.07	43.17	9.8	27.3	30.3	2.49	7.48	1.13	1.28	1.47	0.085
3	2.78	40.57	10.4	23.3	25.9	2.65	9.14	1.27	1.53	2.25	0.088
3.5	3.08	38.72	10.3	20.2	23.9	2.81	9.90	1.25	1.41	5.28	0.105
4	2.72	29.50	10.4	20.5	22.1	3.73	9.85	1.21	1.36	5.60	0.121
5	1.78	22.10	9.4	14.0	14.8	4.92	10.34	1.22	1.13	7.44	0.123

The symbols: under Table 1.

**Table 5:** Mechanical properties of the CR/BR (80/20 by wt %) blends cross-linked with different amount of zinc at 160 °C for 30 min.

Zn (phr)	S_e100_ (MPa)	S_e200_ (MPa)	S_e300_ (MPa)	TS_b_ (MPa)	E_b_ (%)	TS’_b_ (MPa)	E’_b_ (%)	K (-)
1	0.77	1.13	1.51	5.52	456	1.19	39	0.018
2	0.74	1.14	1.62	5.53	509	1.28	47	0.021
2.5	0.67	1.05	1.48	8.60	750	1.39	29	0.006
3	0.60	0.88	1.19	7.80	796	1.67	60	0.016
3.5	0.69	1.24	1.92	7.25	500	1.77	34	0.017
4	0.56	0.90	1.33	7.77	818	1.63	48	0.012
5	0.62	1.02	1.50	4.99	553	1.92	51	0.035

The symbols: under Table 2.

**Table 6:** The cure characteristics and mechanical properties of filled CR/BR/Zn (80/20/2.5 by wt %) compounds, T = 160 °C, t = 40 min.

Fillers	τ_02_ (min)	τ_90_ (min)	M_min_ (dNm)	ΔM_40_ (dNm)	CRI (min^−1^)	Q_v_^T^ (mL/mL)	TS_b_ (MPa)	E_b_ (%)	TS’_b_ (MPa)	E’_b_ (%)
Silica	3.13	37.67	21.2	13.3	2.90	10.14	10.08	656	4.42	30
Kaolin	3.67	38.67	11.6	24.2	2.86	8.72	10.03	666	2.73	16
Chalk	3.97	44.63	11.8	29.4	2.46	6.52	9.62	605	4.53	56
MMT	4.88	35.63	10.9	17.8	3.25	11.56	6.58	717	2.05	19

The symbols: under Table 1 and Table 2.

**Table 7:** Payne effect of unfilled and filled CR/BR/Zn vulcanizates.

Parameter	Unfilled	Silica	Kaolin	Chalk	MMT
ΔG’ (10^3^·Pa)	133.2	1856.8	395.1	427.9	150.4
G’_max_ (10^3^·Pa)	215.1	2045.6	408.1	513.5	343.3
G”_max_ (10^3^·Pa)	32.2	510.5	67.0	130.0	88.0

ΔG′—Payne effect, G′_max_—maximum storage modulus, G″_max_—maximum loss modulus.

**Table 8:** Average values of parameters characterizing the flammability of unfilled or filled CR/BR/Zn (80/20/2.5 by wt %) compounds, determined by cone calorymetry.

Fillers	TTI (s)	THR (MJ/m^2^)	HRR (kW/m^2^)	HRR_max_ (kW/m^2^)	EHC (MJ/kg)	EHC_max_ (MJ/kg)	PML (%)	MLR (g/m^2^·s)	1/t_flashover_ (kW/m^2^·s)
–	54	14.6	76.26	224.23	5.99	60.97	92.7	37.89	4.15
Silica	28	9.5	58.26	92.20	5.45	45.17	59.8	14.58	3.29
Kaolin	43	14.6	123.68	190.48	6.98	62.39	75.1	31.51	4.43
Chalk	76	20.8	130.95	301.53	12.02	71.44	63.0	22.10	3.97
MMT	43	23.3	196.20	414.01	11.06	76.97	97.7	49.99	9.63

TTI—time to ignition, THR—total heat release, HRR—heat release rate, HRR_max_—max heat release rate, EHC—effective heat of combustion, EHC_max_—max effective heat of combustion, PML—percentage mass loss, MLR—average specific mass lost rate, 1/t_flashover—_fire propagation rate.
